# Estimation of Mixed Layer Depth in the Gulf of Aden: A New Approach

**DOI:** 10.1371/journal.pone.0165136

**Published:** 2016-10-27

**Authors:** Abdulla C. P, M. A. Alsaafani, T. M. Alraddadi, A. M. Albarakati

**Affiliations:** 1 Department of Marine Physics, Faculty of Marine Science, King Abdulaziz University, Jeddah, Saudi Arabia; 2 Department of Earth & Environmental Sciences, Faculty of Science, Sana’a University, Sana’a, Yemen; University of California San Diego, UNITED STATES

## Abstract

The mixed layer depth (MLD) in Gulf of Aden is analyzed using vertical high resolution (1m) profiles of both temperature and density. Firstly, we examined threshold and gradient methods for estimating the MLD. Close evaluation with individual profiles reveals the failure of both methods for most of the profiles. Furthermore, the curvature method, a relatively recent approach to define ocean MLDs, is established for open water profiles but for marginal seas, like the Gulf of Aden, it detects shallower depths than the actual MLD. These considerable differences motivated us to introduce a new approach of MLD identification, which is developed based on curvature method and is called segment method. Our segment method produces adequate MLD estimates for more than 95% of the profiles and overcomes major limitations of conventional methods. It is less biased and least scattered compared to other methods with a correlation coefficient > 0.95. The mixed layer in Gulf of Aden displays significant seasonal variability and is deeper in winter. Throughout the year, the western part of gulf experiences deeper mixed layer than the eastern part. Regional eddies dominate Gulf of Aden’s MLD pattern during all seasons.

## Introduction

Continuous energy transfer between atmosphere and ocean develops a quasi-uniform upper layer with nearly uniform temperature, salinity, and density. The depth of this layer (called mixed layer depth or MLD) is important as it determines the volume or mass of water over which flux from the atmosphere is distributed [1–4]. MLD and its variability has been well documented globally [5–7] and regionally [8–12] and has strong impact on near-surface acoustic applications [13], ocean biology [14] and evolution of surface parameters like SST [15].

Previous studies adopted different approaches to identify MLD. The simplest approach is threshold method which is widely used both regionally [3,11,16] and globally [4,6]. Another common approach is gradient method that also is used in small and large scale studies [17–19]. Recently [7] estimated MLD using curvature of the profile. Threshold and gradient methods fix MLD at the shallowest depth where chosen threshold or gradient is achieved. Curvature method searches for the first extreme curvature of the profile, analyzes the profile at nearby levels and defines MLD. [7] visually examined 500 random profiles from various parts of the world and found that estimates from curvature method are better than threshold method for 63% of profiles and vice versa for 10% while for the remaining 27% it is not clear which method is reproducing the adequate MLD.

Gulf of Aden (GA), a marginal sea that connects the Red Sea with the Indian Ocean, augments east-northeastward from the narrow Strait of Bab-el-Mandab to a line interfacing Ras Baghashwa (east of Mukalla, Yemen) and Ras-Asir (northern corner of the Somali Peninsula). It is 900 km long and spreads over an area of around 220 × 103 km^2^ with an average depth of 1800 m, and is strongly influenced by seasonally reversing winds. Circulation and hydrographic changes are largely forced by seasonal changes in wind pattern [20,21]. Compared to other regions of the world, information of MLD and its variability is sparse in the Gulf of Aden. MLDs detected using available conventional (threshold, gradient and curvature) methods are mismatching considerably with each other at the same and adjacent stations. Present work aims to develop a suitable method for MLD estimation in the Gulf of Aden and to understand the seasonal variability. The paper is arranged as follows: First section includes a brief description of mixed layer depth. Second section explains data and methodology used in the present work. Third section describes MLD pattern from conventional methods, difficulties in conventional methods and importance of new approach.

## Data and Methods

### Data set

#### Temperature and salinity profiles

Two hydrographic datasets are used in this study, first is the NODC (National Oceanographic Data Center, http://www.nodc.noaa.gov/OC5/SELECT/dbsearch/dbsearch.html) product of temperature and salinity measured using CTD/STD (conductivity-temperature-depth/salinity-temperature-depth) and second is the REDSOX (Red Sea Outflow Experiment) cruise profiles. About 433 CTD profiles are available in the study area from NODC, out of it 132 belong to winter (Dec-Mar) and 217 belong to summer (Jun-Sep) while remaining belong to inter-seasons. REDSOX experiment provided 238 profiles during winter (Feb-Mar) and 227 during summer (Aug-Sep) in the year 2001 [22,23]. CTD profiles from REDSOX experiment are used to compare MLD identification methods. Seasonal MLD structure is studied using all available CTD profiles described here.

#### Sea level anomaly

Satellite altimetry data (Sea Level Anomalies, SLA) from AVISO (ftp://ftp.aviso.altimetry.fr/global/delayed-time/grids/msla/all-sat-merged/h/) were used to understand sea level changes of the region. Satellite estimates of TOPEX/Poseidon, Jason-1, ERS-1/2, and Envisat are merged together to produce SLA which is available on 0.25x0.25 degree grid from 1992 to present. Detailed information on SLA product and data processing are well documented [24,25]. SLA in the year 2001 is analyzed to see the effect of sea level changes on mixed layer structure.

### MLD estimation

To select best approach for identification of MLD in GA, several methods were applied and a comparison of results from those methods is presented in this section. A short description of each method is presented below.

#### Conventional methods to estimate MLD

Threshold, gradient and curvature methods are applied to identify MLD in the Gulf of Aden. Researchers used different threshold values for both temperature and potential density (here density is used instead of potential density). The most common value is 0.2°C for temperature and 0.03 kg m^-3^ for density. Various thresholds are used from 0.1°C to 1.0°C for temperature and from 0.01 to 0.10 kg m^-3^ for density. [18] reported a suitable value of temperature gradient as 0.025°C m^-1^. [19] used temperature gradient as 0.005°C m^-1^ and potential density gradient as 0.0005 kg m^-3^db^-1^ for Antarctic and sub-Antarctic profiles. [17] used different potential density gradient values ranging from 0.0005 to 0.05 kg m^-3^m^-1^. In this study gradients from 0.005 to 0.05°C m^-1^ are used for temperature and from 0.0005 to 0.03 kg m^-3^ m^-1^ for density. The curvature method identifies MLD with the help of gradient and curvature of profile. MLD is the first maximum of curvature in temperature or density profile with significant gradient at deeper levels [7].

Different methods show significantly different MLD values for the same profile. Similarly substantial differences are observed in MLD of adjacent stations with the same method. Close evaluation of individual profiles revealed ineffectiveness of these methods for a large number of profiles. In the case of profiles where conventional methods detected nearly accurate MLD, curvature method showed better agreement. [7] found that curvature method is better than threshold method for most parts of global ocean. To get a more accurate way for MLD identification, a modified form of curvature method is developed, called segment method. Segment method of MLD estimation and its advantages over other methods are discussed below.

#### Segment method

**Fig 1:**
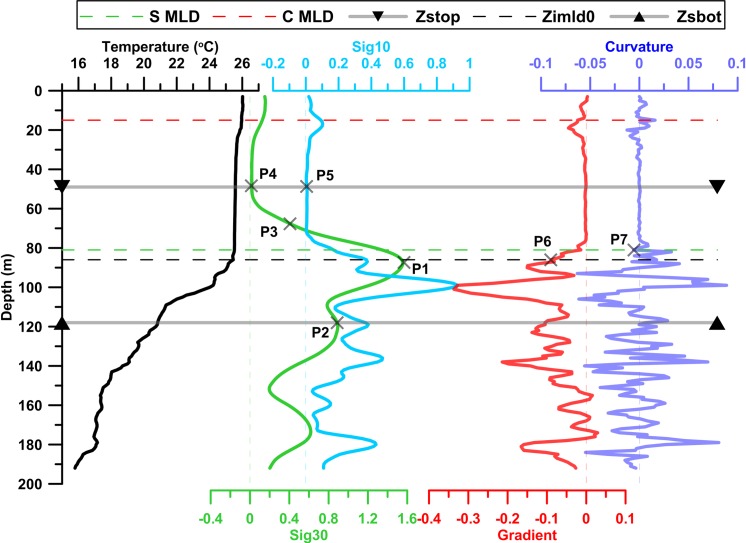
Typical temperature profile to show the procedure of segment method. sig30 (standard deviation for every lower 30 m water column), sig10 (standard deviation for every lower 10 m water column), gradient (with respect to lower 5 m interval) and curvature of temperature are plotted in green, sky-blue, red and blue colors respectively. Horizontal lines with upward and downward pointed triangle marks top and bottom ends of profile segment. Dashed lines represent MLD by curvature method (red), MLD by segment method (green) and first guess MLD (black). Important depths are marked as P1, P2 etc. and are explained in text.

Segment method detects MLD by selecting a portion of profile called “profile segment” which is in between surface and the bottom of thermocline, where the MLD should be identified. Standard deviation and gradient of the variable are used to fix upper and lower limits of profile segment. At first, the bottom and top end of profile segment are calculated. Then the closest level to MLD is fixed by analyzing the profile segment. Detailed explanation of procedure based on temperature profile (*T*) is given below:

**a) Identifying profile segment.** Profile segment, a portion of the profile within the surface and thermocline, is fixed as follows:

Gradient (*g*_*T*_) with respect to a level 5 m deeper and curvature (*c*_*T*_) are calculated at each level as in [7]. σ_30_ is the standard deviation of T over the levels in a 30 m interval below the current level (the current level also included). σ_10_ is defined similar to σ_30_, but for deeper 10 m interval. σ_30_ and σ_10_ represent the homogeneity of the profile. Following [7], and analyzing profiles at various parts of the study region, a profile is assumed to have significant variability if σ_30_ exceed 0.02 at any depth, and then the profile is considered for MLD estimation. Fig 1 shows a typical profile of the region and MLD identification procedure. Identification of profile segment consists of two parts as describe below.

Local maxima of (σ30) represent the regions of strong gradients. Maximum of σ_30_ (σ_30max_) is usually located at or near thermocline (denoted by P1 in Fig 1). The bottom end of profile segment, *Z*_*sbot*_ is the level 30 m deeper to the level of σ_30*max*_ (denoted by P2 in Fig 1). Starting point of the homogeneous layer of the profile segment is called as *Z*_*stop*_. For ideal profiles, *Z*_*stop*_ will be first depth near to the surface. But for profiles with short term intrusion (or gradient) within the mixed layer, the very first depth after short term intrusion is considered as *Z*_*stop*_. To fix *Z*_*stop*_, derived variables σ_30_ & σ_10_ are used. The first occurrence where $\sigma_{30}\geq \frac{1}{4}*\sigma_{30max}$ is found (denoted by P3 in Fig 1). Minima in σ_30_ curve represents nearly homogeneous levels of water. Initially, the local minimum which is shallower and nearest to P3 is identified (denoted by P4 in Fig 1). To confirm homogeneity of the region, the variability of σ_10_ is analyzed in the lower 10 meters of water. Depth at which σ_10_ is very low (less than 5% of σ_30max_) with a minimum value of σ_10_ is considered as Z_*stop*_ (denoted by P5 in Fig 1). The portion of profile between *Z*_*stop*_ and *Z*_*sbot*_ is named as “profile segment”.

**b) Analyzing the profile segment and identifying the level closest to MLD (*Z***_***imld***_**)**. The profile is analyzed from *Z*_*stop*_ to bottom to find the closest level to MLD. At First, the shallowest depth where |*g*_*T*_(*i*)| > 0.25 * *max*|*g*_*T*_| and σ30(i) > 0.02 is identified (represented as *Z*_*imld*0_ and denoted by P6 in Fig 1). The second criterion makes sure that, estimated MLD is at a location with significantly inhomogeneous deeper levels. Usually *Z*_*imld*0_ is found at shallower end of the thermocline and below MLD.

Standard deviation of *g*_*T*_ at interval [*Z*_*stop*_,*Z*_*imld*0_], (denoted as $\sigma_{g_{t}}$) denotes range of variability in the interval. Following [7], closest level to MLD (*Z*_*imld*_, also denoted by P7 in Fig 1) is the shallowest depth where minima/maxima of the curvature falls together with positive/negative gradient *g*_*T*_. In addition to this, two conditions are also applied to confirm MLD. Firstly $|g_{T}|>\sigma_{g_{t}}$, which assures a threshold for significant local inhomogeneity in the profile. Secondly σ_30_(i) > 0.02, that confirms that the level identified is above the region of rapid changes. For low resolution profiles, it is recommended to apply interpolation to get more precise MLD. Interpolation process applied in [7] is used in this study. If no extreme value is found in the profile segment, then the first level where |*g*_*T*_| ≥ 0.7 * *max*|*g*_*T*_| is considered as MLD. Such MLDs are flagged in the algorithm. None of the profiles of both winter and summer season used in this study faced this situation. A flowchart showing the steps of MLD estimation procedure is given in the supporting information section (S1 File).

## Results and Discussion

### Mixed layer depth based on conventional methods

**Fig 2:**
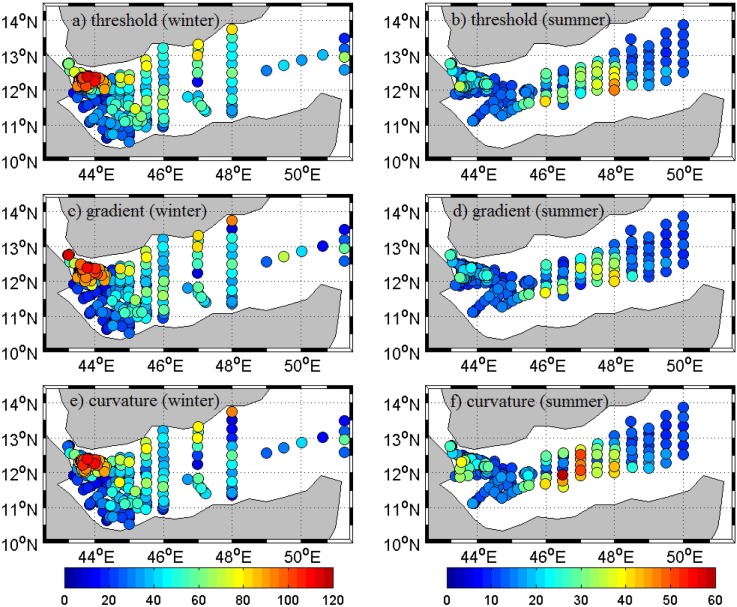
Temperature based MLD profiles during winter (left panel) and summer (right panel) based on conventional methods. Threshold, gradient and curvature based maps are shown in top (a & b), middle (c & d) and bottom (e & f) panels respectively. Scale for left and right panels are kept separate for better understanding of differences.

Conventional methods are used to identify MLD of the region in both winter and summer using temperature and density profiles from REDSOX experiment. Fig 2 shows the estimated MLD using threshold (with common threshold criteria for temperature, 0.2°C), gradient (with common gradient criteria 0.025°C) and curvature method.

Estimated mean MLD using common temperature threshold (Fig 2A and 2B) during winter and summer are respectively 35 m and 15 m, with minimum 11 m (10 m) and maximum 102 m (39 m) in winter (summer). Temperature and density profiles are used in the analysis and the results for both are similar. Hereafter, if not specified, statistical parameters like mean, maximum, bias, correlation coefficient, etc. are explained based on temperature profile only. Fig 2C and 2D shows estimated MLD in the region using the gradient method with a gradient of 0.025°C. Obtained mean MLD with gradient approach is 65 m (22 m) in winter (summer), with minimum 29 m (10 m) and maximum 116 m (50 m). MLD based on curvature method (Fig 2E and 2F) show mean MLD as 47 m (20 m) with the minimum at 12 m (10 m) and maximum at 111 m (47 m) in winter (summer). MLD values based on threshold, gradient and curvature methods differ from each other at many locations.

### Mixed layer depth based on segment method

**Fig 3:**
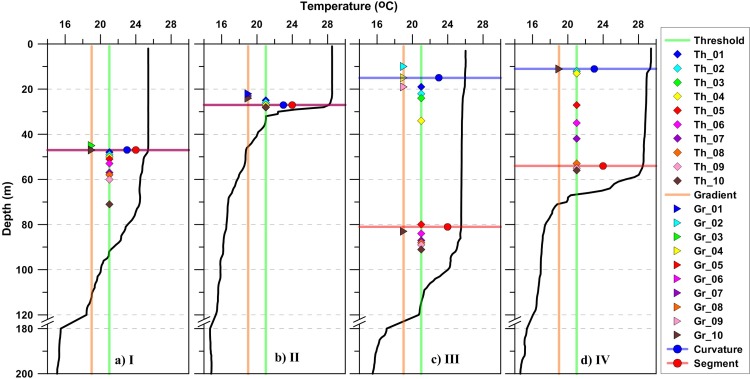
Profiles marked with MLD based on different methods for four sample stations I, II, III and IV. I, III are winter profiles and II, IV are summer profiles. Threshold (Gradient) MLDs are marked on green (orange) vertical line. Curvature (segment) method based MLD is marked by horizontal line with a dot in blue (red) color. Letters “Th” and “Gr” are used in labels to represent threshold and gradient methods. Numbers in the tail of label indicate used threshold (0.1, 0.2, 0.3, 0.4, 0.5, 0.6, 0.7, 0.8, 0.9, and 1.0 respectively) and gradient (0.005, 0.01, 0.015, 0.02, 0.025, 0.03, 0.035, 0.04, 0.045, and 0.05 respectively).

Above approaches (Fig 2) showed considerable differences in estimated MLD in both winter and summer. Close observation of individual profiles and corresponding MLD values revealed the limitations of each method. Temperature based estimates of MLD using threshold, gradient, curvature and segment based approaches for four sample stations are shown in Fig 3. Profile I & III are during winter and II & IV are during summer.

MLD for the station I, using threshold approach is approximately between 50 to 70 m, and with gradient method is around 45 m. It is interesting that both curvature and segment methods detect MLD at 48 m. MLD observed at station II with all threshold and gradient criterions are between 20 to 30 m while curvature and segment methods detect at the same depth.

In the case of profile at station III, for lower criterions threshold method and gradient method define MLD between ~20 to ~30 m while at ~90 m for the remaining. Curvature method defines at 15 m and segment method defines at 81 m. Segment method based MLD is nearly five times greater compared to curvature based MLD. For Profiles at station IV, threshold method detects between ~10 m to ~55 m while almost all of the gradient criterions detect MLD around 11 m. Curvature method defines MLD at 11 m for temperature, whereas segment method defines at ~55 m. Considering profiles at stations I and II, it is to be noted that most of the criterions detect MLD at nearby (<5 m difference) levels, which implies these are applicable for profiles having nearly ideal structure. But in the case of profiles at stations like III and IV, detected MLD by different methods has a substantial difference from one another. For some profiles, such differences are many times larger than the other.

### Differences and limitations of conventional methods

**Fig 4:**
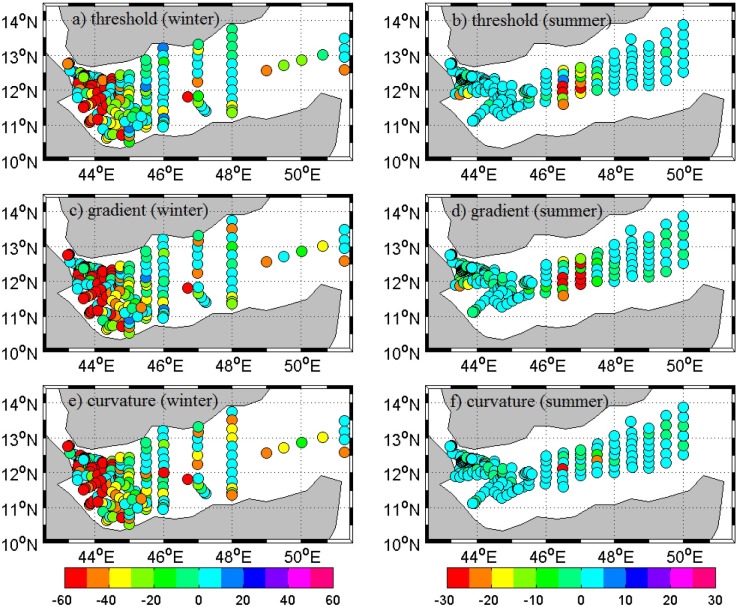
Difference between conventional and segment methods based MLD estimates for profiles during winter (left panel) and summer (right panel). Threshold, gradient and curvature maps are shown in top (a & b), middle (c & d) and bottom (e & f) panels respectively. Scale for left and right panels are kept separate for better understanding of differences.

The differences in estimated MLD between conventional methods and segment method are shown in Fig 4. Difference between MLDs shows spatio-temporal variability at most of the stations. Number of stations having higher difference is more in winter than in summer. Most of the stations at the western part of gulf experienced large differences. MLD estimates based on curvature method show small differences at relatively large number of stations, especially during summer. Generally, higher extreme criterions showed overestimation while lower extremes resulted in underestimation (Fig 3).

To analyze the performance of MLD estimation, [7] selected 500 profiles from various parts of the world and manually compared their method to threshold method. Similarly best MLD from four methods (threshold, gradient, curvature, and segment), are compared with a visually defined MLD (fixed by manual observation of each profile, hereafter VMLD). VMLD is the bottom of visibly quasi-homogeneous upper layer with a rapidly varying lower water column. Quality index (described in the next section) is used to confirm the reliability and accuracy of VMLD.

Number of stations available in each grid has a significant spatial difference with relatively higher number of stations in western part of study area (Fig 2). Statistical analysis for all profiles may represent the region with the higher number of profiles (west GA). Keeping this in mind, randomly selected one profile for every 0.25°*0.25° bin and are used for comparison.

#### Quality index

**Fig 5:**
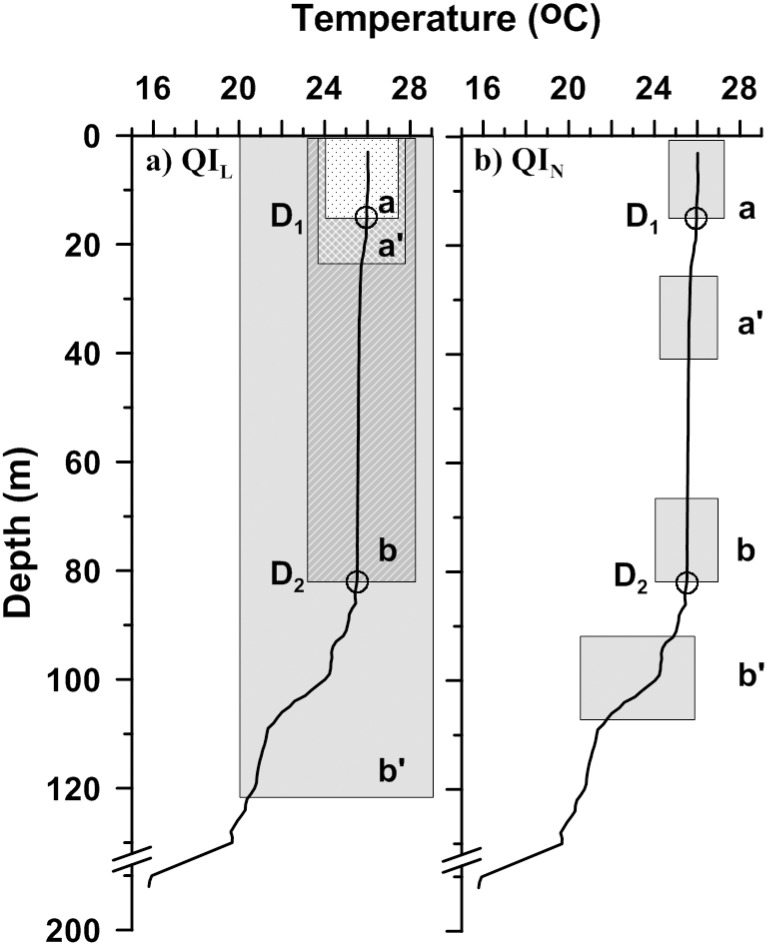
**Schematic diagram to explain quality index using (a) QI_L_ and (b) QI_N_.** D_1_ and D_2_ are two arbitrary depths to check performance of QI. Shaded boxes are labeled as a & a’ and b & b’, representing the portion of profile used to calculate standard deviation at D1 and D2 respectively.

Quality index is prepared based on the notion that MLD is the bottom of nearly-homogeneous surface layer followed by a rapidly varying lower layer. Standard deviation of the variable from surface to MLD is expected to be nearly zero and that of deeper levels substantially high. [7] estimated quality index at arbitrary depths D1 and D2 (Fig 5A) as:

$${QI}_{L}=1-\frac{c}{c^{\prime }}=1-\frac{\sigma ({T_{i}-T_{mean})|}_{\;(z1,\;MLD)})}{\sigma ({T_{i}-T_{mean})|}_{\;(z1,\;1.5*MLD)})}$$ (1)

where σ denotes standard deviation with respect to vertical mean from nearest surface depth (z_1_) to MLD or 1.5*MLD. Letters c & cʹ (portion of the profile used to calculate σ) represented by a & aʹ at D1 and b & bʹ at D2. Based on QI_L_, quality of MLD is categorized into three: 1-“well-defined” (QI_L_ > 0.8), 2-“uncertainty present” (QI_L_ between 0.5 and 0.8) and 3-“no direct interpretation possible” (QI_L_ < 0.5).

QI_L_ has been applied on profiles to get the accuracy of MLD estimation. It has been found that QI_L_ satisfactorily estimates the quality of MLD for most of the profiles used in the study. In some cases where the profiles have short range gradient within the mixed layer, QI_L_ is found to have higher value for bad MLD estimates also. To overcome this limitation a new (additional) quality index is introduce in this study. The additional quality index (QI_N_) is defined at arbitrary depths D1 and D2 (Fig 5B) as:

$${QI}_{N}=1-\frac{c}{c^{\prime }}=1-\frac{\sigma ({T_{i}-T_{mean})|}_{\;(MLD-15m,\;MLD)})}{\sigma (T_{i}-T_{mean}){|}_{\;(MLD+10m,\;MLD+25m)})},$$ (2)

where σ is calculated for 15 m water column just above (*b*) and 10 m below (*b*′) of MLD as shown in Fig 5B. A 10 m gap is kept between *b and b*′ to keep away the short range gradient (if any present) from calculation. Fig 5 shows schematic diagram of quality index calculation at two arbitrary depths, which selected to compare the performance of quality index, a very shallow depth (D_1_ at 15m) and a more realistic depth where MLD is expected (D_2_ at 82m).

The corresponding values of QI_L_ and QI_N_ at depth D1 are 0.7 and 0.21 while at depth D2 are 0.91 and 0.99. The values of QI_L_ and QI_N_ are high at depth D2, indicating good quality of MLD estimation. But at D1, QI_L_ is relatively high (close to 0.8) and QI_N_ is very small, where small values are expected. The unexpected high value of QI_L_ is due to the presence of short range gradient at depth D1. Quality of MLD estimation is determined by considering both QI_L_ and QI_N_. QI_N_ < 0.8 indicates the presence of inhomogeneity in the upper layer. If both QI_L_ and QI_N_ are ≥ 0.8, then defined MLD assumed to be “well-defined”. The values of quality index and corresponding quality category are tabulated in Table 1.

**Table 1:** Quality category and corresponding values of QI_L_ and QI_N_.

	QI_N_	QI_L_	Quality category
1	≥0.8	≥0.8	Well defined
		0.5–0.8	Acceptable MLD
		<0.5	No direct interpretation possible
2	0.5–0.8	≥0.8	Uncertainty present
		<0.8	No direct interpretation possible
3	<0.5	Any	No direct interpretation possible

Out of the VMLD defined profiles, 86% come under the well-defined category with QI_L_ ≥0.8 and QI_N_ ≥0.8, while the rest have QI_L_ ≥0.7 and QI_N_ ≥0.8. VMLDs that come under well-defined category are only used for comparison, to guarantee higher accuracy and reliability on manually defined VMLD.

#### Comparison of methods

The difference between VMLD and method based MLD is analyzed over the region. Since the mixed layer is thicker in winter than summer, difference between VMLD and method based MLD are larger in winter than in summer. Analyses discussed in this section are based on winter profiles only. Summer profiles also produced similar results, but are weaker than those in winter.

**Fig 6:**
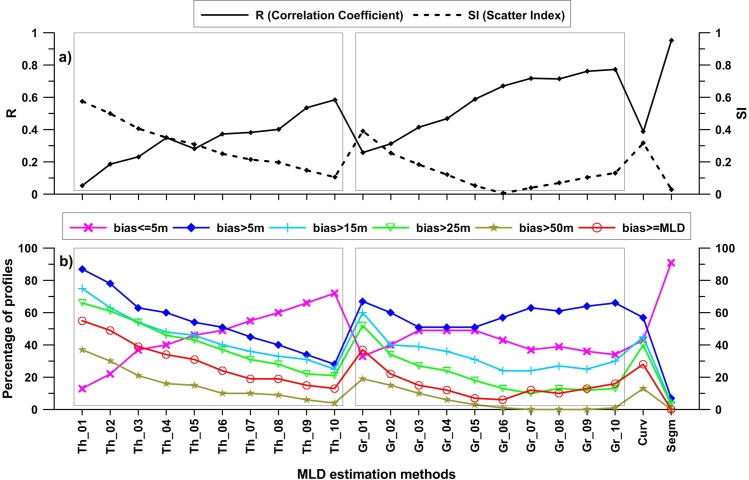
Statistical parameters calculated for threshold, gradient, curvature and segment methods with respect to VMLD. Two boxes are drawn in both figures to represent different threshold (left box) and gradient (right box) criterions. R (correlation coefficient) and SI (Scatter index) are plotted in top panel. Bias is plotted in bottom panel. “Th” in the label represents threshold method and the number given at tail of each label denotes the respective criterion (similarly “Gr” for gradient method). “Curv” and “Segm” represent curvature and segment methods respectively.

Statistical parameters computed for threshold, gradient, curvature and segment based approaches against VMLD are shown in Fig 6 and described below. Temperature thresholds varying from 0.1°C to 1.0°C were used. All of the selected thresholds are found to be significantly biased. Common temperature threshold used in threshold method is 0.2°C, which showed very weak correlation (Fig 6A). About 61% of profiles showed 25 m or more bias, of them 30% have >50 m bias. For 49% of profiles, the detected MLD is just half or less than that of VMLD. Higher thresholds examined also failed to identify realistic MLD in many cases with overestimation. For a threshold of 1.0°C (the highest of the selected thresholds and 5 times greater than commonly used 0.2°C), approximately 25% and 21% of profiles show a bias of 15 m and 25 m respectively. All of the thresholds are weakly correlated with VMLDs. Lower thresholds are well scattered (SI > 0.5) and scattering gradually decreased to higher threshold end (SI < 0.2). Observed bias is greater than the detected MLD itself for 50% of profiles at lower thresholds and close to 20% at higher thresholds.

Threshold method is strongly depended on chosen criterion. As threshold becomes larger, detected mixed layer becomes deeper (Fig 3D). In conditions where the vertical gradient is low, detected MLD for different thresholds turn out to be significantly separated to each other and stickier in the opposite case. Similarly for two profiles with similar mixed layer, depending on the surface property value (for example SST) the identified MLD can be different [7]. Profile with lower SST will show deeper MLD in such condition. Compared to segment method, threshold method has a poor estimation of MLD.

Gradient method has strong bias with respect to VMLD at lower gradients and becomes weaker at higher gradients. Gradient 0.03°C is the least scattered among examined 10 gradients with a correlation of 0.66. Even though, at this gradient, 24% of profiles have a minimum 15 m bias. For the commonly used gradient (0.025°C), 31% have a 15 m difference with low correlation (0.58). Gradients ≥ 0.03°C are relatively better correlated to VMLD estimates, but nearly 30% of profiles show at least 15 m bias for all gradient thresholds. The analysis shows gradient method is better than threshold method, but still has considerable weaknesses.

Similar to threshold method, gradient method also shows strong dependence on chosen gradient criterion. Other than that, in regions with small intrusion or short scale gradient in the profiles, gradient method detects MLD at such depths in most cases, which leads to large differences between detected and actual mixed layer. It strongly indicates the ineffectiveness of gradient method for GA region.

In curvature method, 40% of profiles show a bias of 25 m or higher while about 13% show 50 m bias. For nearly 28% of profiles, the detected MLD is just half of or lesser than that of VMLD. Curvature method is weakly correlated (0.38) to VMLD estimates. Dynamically active regions may possess short range gradients within the mixed layer itself. In such circumstances, curvature method identifies MLD at short gradient depths, which often leads to early detection of MLD.

MLD estimates based on segment method have strong correlation (R = 0.95) and least scattering (SI = 0.02). Gradient criterion 0.03°C also has similar value for SI but shows significant bias. In the case of segment method, among the whole used profiles only 4% has a bias of 15 m or more. Detected bias for 91% of profiles is 5 m or less. Out of the 22 methods used in this study (10 based on threshold, 10 based on gradient, 1 based on curvature and 1 based on segment), segment method is the least biased, least scattered and best correlated.

Segment method detects MLD at realistic depth and is quite helpful in avoiding short range gradients or small scale intrusions, which are present in many profiles. Similar to curvature method, segment method is free from dependence on property value at the reference depth, it is quite easy to implement to any region. For methods like threshold and gradient, it is necessary to change chosen criterion with characteristics of the region. Having no such requirement and its ability to overcome short range gradient makes segment approach more acceptable.

In the case of profiles having ideal structures, with no significant small scale gradients within the mixed layer, all the methods identify nearly equal MLDs (Fig 3B). Many of profiles in GA have short range gradient at near surface depth with quite uniform characteristic layer beneath, followed by a clearly visible thermocline. For this reason segment based approach is used for MLD estimation in GA.

### MLD pattern in the Gulf of Aden

**Fig 7:**
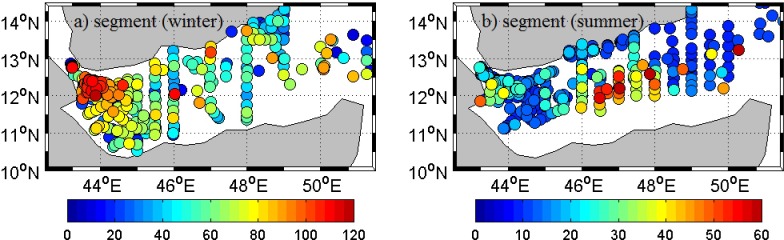
**Map of MLD identified using temperature based on segment method for available CTD profiles during winter (a) and summer (b).**

Segment method is used to define MLD in the region using all available temperature profiles from CTDs during winter (Dec-Mar) and summer (Jun-Sep) months, shown in Fig 7. Maximum MLD over the region during winter (summer) is 120 m (60 m) at far west (central part) and the minimum is 22 m (10 m) at far east (west and east) with mean 77 m (21 m).

**Fig 8:**
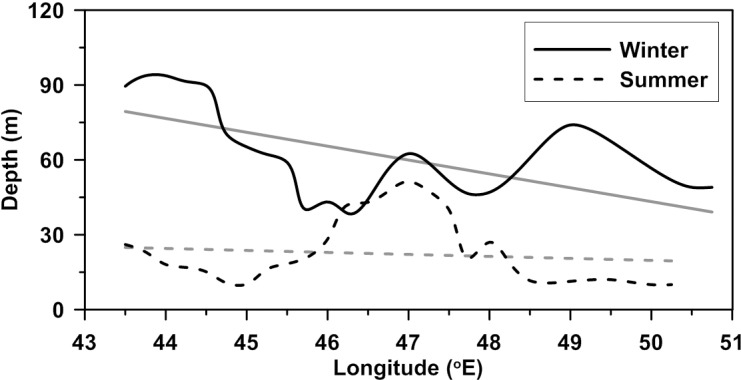
MLD along the central axis of gulf extending from west to east. Thick solid line (thick dashed line) represent MLD during winter (summer). Solid and dashed thinner lines represent linear fit to winter and summer MLD respectively.

During winter, western GA has deeper mixed layer and becomes shallower to the eastern side (Figs 7 and 8). Mean MLDs in western, central and eastern parts of gulf are 83 m, 57 m and 49 m respectively. Shallowing tendency of MLD towards east exists in summer also, but is weak (mean MLDs are 20 m, 42 m and 17 m in west, central and east respectively). The mixed layer is shallow at western and eastern part of the gulf with deeper mixed layer at central part. Average MLD calculated along the axis of GA (along the straight line from 11.75N & 43E to 13.25N & 51.5E and meridionally averaged for +/- 0.5 degrees) for winter and summer months of the year 2001 (the year with the largest number of observation) is shown in Fig 8. MLD along the central axis follows the general spatial pattern with higher values towards west and lower towards east.

**Fig 9:**
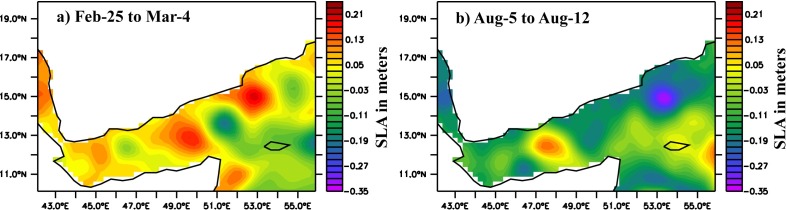
**SLA (in meters) from AVISO for a) 25-Feb to 04-Mar and b) 05-Aug to 12-Aug in 2001.**

GA experiences frequent cyclonic and anti-cyclonic eddies. [26] found the presence of three eddies, two cyclonic and one anti-cyclonic using ADCP current measurements. Sea Level Anomalies from AVISO are shown in Fig 9 for two days respectively in winter and summer. [27] confirmed the westward movement of eddies in the region. Multiple numbers of eddies and movement towards the west lead to complex dynamics in the region. During winter, south of the western GA has relatively shallower (~75 m) MLD than that of north (~110 m), which can be related to observed cyclonic eddy at south of the western part (Fig 9A). Similarly, east of 48°E has deeper MLD than the surrounding region. Presence of the anti-cyclonic eddy centered at 49°E might have deepening effect on MLD of the region. Cyclonic eddy at central part of the gulf also showed its signature in the MLD pattern with lower MLD values. In summer, MLD gradually becomes shallow from west to east, with abnormally deeper MLD at central part of the gulf (Fig 8). An anti-cyclonic eddy has existed at central gulf from June (figure not shown). This eddy intensified during July and continued until middle of August (Fig 9B), which might have significantly influenced mixing in the region and resulted in a deeper mixed layer.

## Conclusions

MLD detected by conventional methods is analyzed in Gulf of Aden region. Threshold and gradient methods applied on temperature profiles with commonly used criterion 0.03 kg m^-3^ and 0.005 kg m^-3^m^-1^, underestimate MLD by 20 m for ~50% of profiles in the study area. Lower and higher extreme criterions used also failed to capture reliable depth of mixed layer with under or over estimation. For profiles with more or less ideal shape, all techniques estimated MLD with an acceptable difference of <5 m. In such conditions, curvature method exhibited higher efficiency than threshold and gradient methods. For profiles with irregular shape, conventional methods are unable to identify realistic MLD due to the presence of short range gradients within the mixed layer itself. Segment method which is a new method of MLD estimation is introduced which overcomes major limitations of conventional methods. Curvature and segment methods have two key advantages over threshold and gradient method, i.e. they are independent of the property value at the surface like SST, and estimated MLD is free from linear dependence on the used threshold or gradient criterion. In addition, segment method overcomes limitations of short-range gradient or small scale intrusion that may be present in highly dynamic regions. These advantages of segment method make it more reliable and acceptable. Quality index definition used in this study is useful to confirm the accuracy and reliability of estimated MLD.

Detected MLD by segment method is used to show temporal and spatial variability of mixed layer structure. Generally, in both, winter and summer, the western part of gulf has deeper mixed layer and shallows gradually to the east (Figs 7 and 8). SLA of the region confirms the presence of cyclonic and anti-cyclonic eddies in the region. Eddies in the region influence water up to 1000 m and more [26], and play significant role in mixed layer changes of the region. Deeper mixed layer at the central part of gulf during summer, against the general pattern, is due to the relatively strong anti-cyclonic eddy in the region during this period (Fig 9B). Due to complex nature of the region, detailed investigation on the influence of eddies and other parameters are essential to formulate a clear picture of dynamics associated with MLD changes.

## Supporting Information

S1 File Flowchart showing the steps of MLD estimation procedure. (PDF)
