# Correction: Estimation of Mixed Layer Depth in the Gulf of Aden: A New Approach

**DOI:** 10.1371/journal.pone.0173545

**Published:** 2017-03-02

**Authors:** C. P. Abdulla, M. A. Alsaafani, T. M. Alraddadi, A. M. Albarakati

The first author's name is incorrect in the citation and the byline. The correct name is: C. P. Abdulla.

The correct citation is: Abdulla CP, Alsaafani MA, Alraddadi TM, Albarakati AM (2016) Estimation of Mixed Layer Depth in the Gulf of Aden: A New Approach. PLoS ONE 11(10): e0165136. doi:10.1371/journal.pone.0165136
