# Supplementary figures and images for: Estimation of Mixed Layer Depth in the Gulf of Aden: A New Approach
Source: PLoS One. 2016 Oct 27;11(10):e0165136. doi: 10.1371/journal.pone.0165136 (PMC5082926; doi:10.1371/journal.pone.0165136)

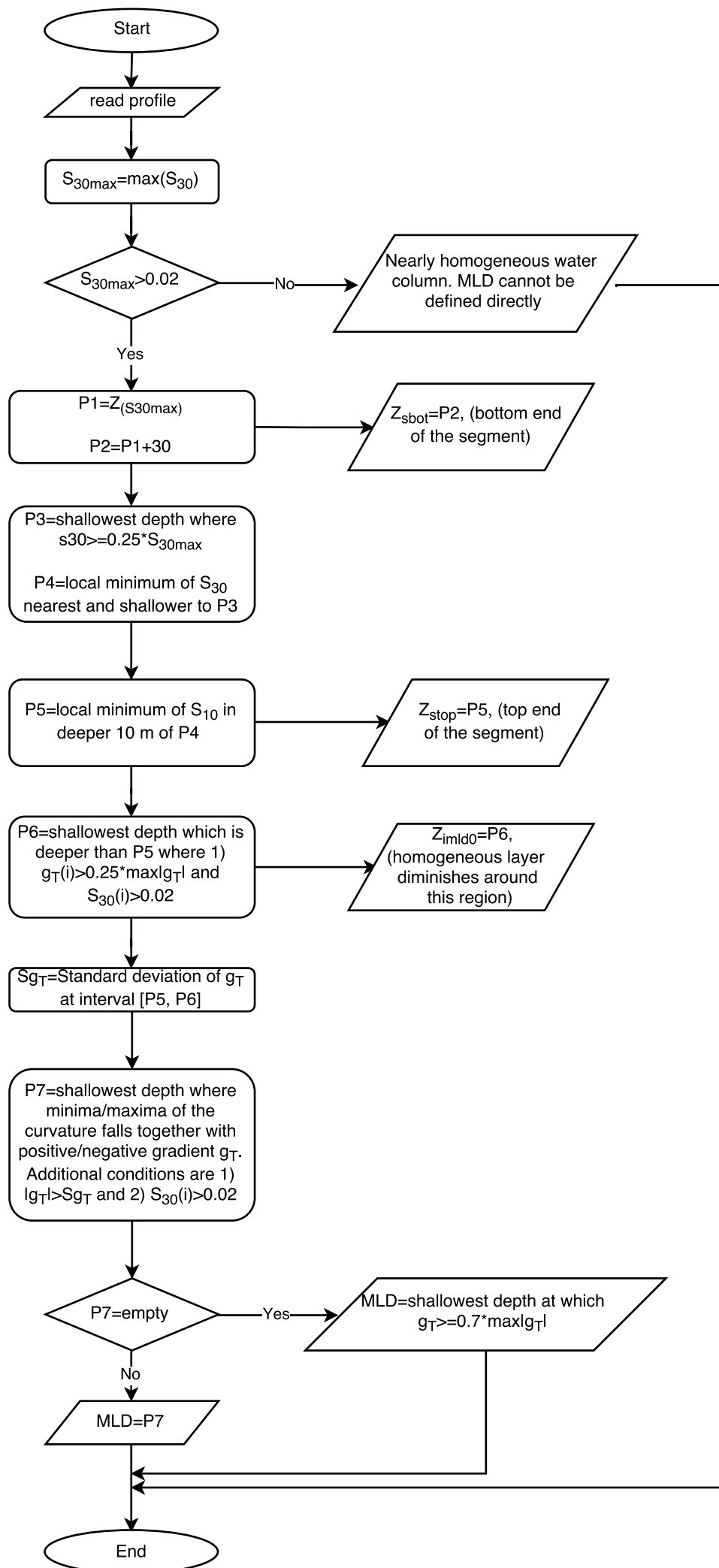

Supplement: S1 File — (PDF) [file pone.0165136.s001.pdf]
